# The Molecular Bases of Anti-Oxidative and Anti-Inflammatory Properties of Paraoxonase 1

**DOI:** 10.3390/antiox13111292

**Published:** 2024-10-25

**Authors:** Hieronim Jakubowski

**Affiliations:** 1 Department of Biochemistry and Biotechnology, University of Life Sciences, 60-637 Poznań, Poland; jakubows@rutgers.edu; Tel.: +1-973-972-8733; Fax: 973-972-8981; 2 Department of Microbiology, Biochemistry and Molecular Genetics, International Center for Public Health, New Jersey Medical School, Rutgers University, Newark, NJ 07103, USA

**Keywords:** PON1 physiological substrates, homocysteine thiolactone, 5-(3′,4′-dihydroxyphenyl)-γ-valerolactone, PON1 proteomics, anti-oxidant proteins, anti-inflammatory proteins, cardiovascular disease, Alzheimer’s disease, cancer

## Abstract

The anti-oxidative and anti-inflammatory properties of high-density lipoprotein (HDL) are thought to be mediated by paraoxonase 1 (PON1), a calcium-dependent hydrolytic enzyme carried on a subfraction of HDL that also carries other anti-oxidative and anti-inflammatory proteins. In humans and mice, low PON1 activity is associated with elevated oxidized lipids and homocysteine (Hcy)-thiolactone, as well as proteins that are modified by these metabolites, which can cause oxidative stress and inflammation. PON1-dependent metabolic changes can lead to atherothrombotic cardiovascular disease, Alzheimer’s disease, and cancer. The molecular bases underlying these associations are not fully understood. Biochemical, proteomic, and metabolic studies have significantly expanded our understanding of the mechanisms by which low PON1 leads to disease and high PON1 is protective. The studies discussed in this review highlight the changes in gene expression affecting proteostasis as a cause of the pro-oxidative and pro-inflammatory phenotypes associated with attenuated PON1 activity. Accumulating evidence supports the conclusion that PON1 regulates the expression of anti-oxidative and anti-inflammatory proteins, and that the disruption of these processes leads to disease.

## 1. Introduction

Atherosclerosis is the main cause of morbidity and mortality in the Western world. It is a multifactorial chronic inflammatory disease that involves a complex interaction of circulating cells and blood factors with the blood vessels. The disease starts with endothelial dysfunction, which leads to accumulation of oxidized lipids in the artery wall [1,2]. Lipid oxidation plays a central role in atherogenesis [3] by inducing a pro-inflammatory phenotype in the arterial wall that underlies the development and progression of atherosclerosis [4].

High-density lipoprotein (HDL) is an established protective factor against atherosclerosis due to its ability to mediate reverse cholesterol transport as well as anti-oxidative, anti-inflammatory, and endothelial protective functions [4,5,6]. HDL can inhibit endothelial cell adhesion molecules, such as the vascular cell adhesion molecule 1 (VCAM-1), the intercellular adhesion molecule-1 (ICAM-1), and E-selectin, that enable monocytes to bind at the sites of developing atherosclerosis [7]. HDL can remove peroxidized lipids from LDL and, subsequently, reduce them in a reaction with methionine residues of apolipoprotein A1 (APOA1) [8]. Lipid-free APOA1 can also remove lipid peroxide molecules from low density lipoprotein (LDL) [9]. Reconstituted HDL containing only APOA1 and phospholipids inhibits LDL oxidation like the native HDL3b and HDL3c particles do [8]. Other HDL-associated lipoproteins and enzymes, including paraoxonase 1 (PON1), have also been implicated in HDL’s anti-oxidative, anti-inflammatory, and endothelial protective functions [10,11].

PON1, a hydrolytic enzyme that requires calcium for activity, is expressed in the liver, kidney, colon [12], and brain [13,14,15], and circulates attached to HDL in the blood. It is a minor HDL protein with potential clinical significance. Proteomic studies revealed that HDL particles carrying PON1 are enriched in several other important proteins such as A2M, ALB, CLU, IGHG1, IGLC2, PROS1, and TF [16].

Studies over the last decade have significantly expanded our knowledge regarding the natural substrates of PON1 and their role in human disease. Other studies have shown that the protective function of PON1 in human health is due to the ability of PON1 to affect the expression of genes involved in anti-oxidative and anti-inflammatory processes. These studies are discussed in the present review, highlighting the involvement of reduced PON1 expression/activity in the pro-oxidative, pro-atherogenic, pro-amyloidogenic, and pro-cancerogenic phenotypes.

## 2. Hydrolytic Activities of the PON1 Enzyme

**Figure 1:**
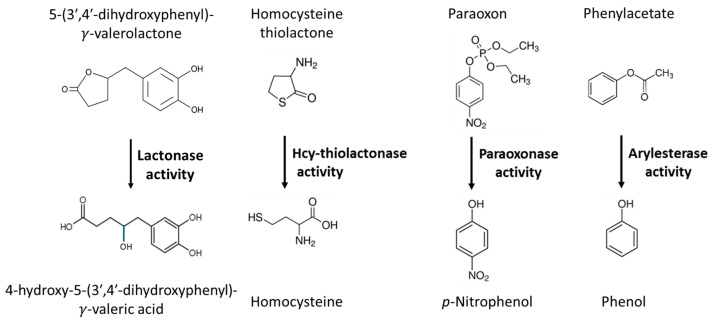
Hydrolytic activities of the PON1 enzyme. Hcy-thiolactone [19,23,24,25] and 5-(3′,4′-dihydroxyphenyl)-*γ*-valerolactone [41] are biological substrates of PON1; paraoxon and phenylacetate are nonbiological substrates of PON1 [17,18].

The *PON1* gene is located on the long arm of chromosome 3 in the *PON* cluster together with *PON2* and *PON3* genes. Its polymorphic variants include *PON1-Q192R* [17], which involves the glutamine (Q) to arginine (R) change at position 192 of the amino acid sequence of the PON1 protein and affects its hydrolytic activity. Historically, the hydrolytic activity of PON1 has been assayed with non-natural substrates such as the organophosphate paraoxon (for which the PON1 enzyme has been named) and phenyl acetate [18] (Figure 1).

Studies of homocysteine (Hcy) metabolism have led to the discovery that PON1 is responsible for the enzymatic hydrolysis of Hcy-thiolactone to Hcy (Figure 1) in human serum, thus identifying the first natural substrate of PON1 [19]. Hcy-thiolactone, a cyclic chemically reactive thioester, is a product of Hcy editing by methionyl-tRNA synthetase during protein biosynthesis [20,21,22].

PON1 is the only Hcy-thiolactone hydrolyzing enzyme in the human blood [19,23]. The Hcy-thiolactonase activity of the PON1 enzyme shows an interindividual variability of over 10-fold [24,25], similar to the interindividual variability in the paraoxonase activity [17]. This variability is mostly due to polymorphisms in the human *PON1* gene [17]. For example, the *PON1-192RR* variant exhibits high activity while *PON1-192QQ* variant has low activity towards Hcy-thiolactone [23] and paraoxon [17]. In contrast, the *PON1-Q192R* polymorphism has an opposite effect on the arylesterase activity: The *PON1-192RR* variant exhibits low arylesterase activity while the PON1-192QQ variant has high arylesterase activity [16,26,27,28,29]. Individuals who have the low-activity *PON1-192QQ* polymorphic variant produce significantly more Hcy-thiolactone than those who have the high-activity *PON1-192RR* polymorphic variant [26].

Low PON1 expression/activity is accompanied by increased oxidative stress and predicts adverse outcomes in cardiovascular disease (CVD) [28,30], diabetes [31,32], neurological disease [33], and cancer [34]. This has been suggested to be due to the mediation by PON1 of anti-oxidative and anti-inflammatory effects of HDL [35,36]. Many other HDL components have also been shown to mediate the anti-oxidative activity of HDL [10,11,37], including APOA1, which accounts for 70% of the HDL protein mass [37] and anti-apoptotic activity [38] and most of the HDL anti-oxidative activity [8]. Accumulating evidence suggests that influence of PON1 on oxidative stress and inflammation is indirect rather than direct [39].

**Figure 2:**
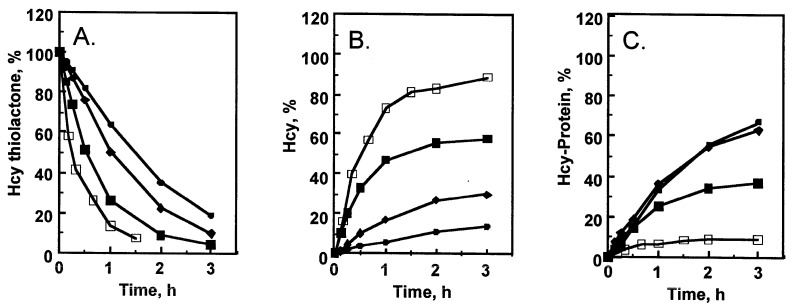
Turnover of Hcy-thiolactone and accumulation of *N*-Hcy-protein in serum at 37 °C. Changes in Hcy thiolactone (3.5 µM at time zero) (**A**), Hcy (**B**), and *N*-Hcy-protein (**C**) levels in human serum from individuals with *PON1-LL55/RR192* genotype (■, t_1/2_ = 0.5 h), *PON1-MM55/QQ192* genotype (♦, t_1/2_ = 1 h), human serum with the PON1 enzyme inactivated by 5 mM EDTA/2 mM D-penicillamine (●, 1.5 h), and rabbit serum (□, 0.25 h). Reproduced with permission from ref. [24]**.** Copyright 2001 by John Wiley and Sons.

Hcy-thiolactone is harmful because it reacts with the ε-amino group of protein lysine residues, forming *N*-homocysteinylated-proteins, which impairs protein’s structure and function [22]. Hydrolytic detoxification of Hcy-thiolactone by PON1 is beneficial because it prevents protein damage by *N*-homocysteinylation [19,24,40]. For example, serum from donors with the *PON1-LL55/RR192* genotype hydrolyzed Hcy-thiolactone (Figure 2A) to Hcy (Figure 2B) faster and afforded better protection from protein *N*-homocysteinylation than serum from donors with the *PON1-MM55/QQ192* genotype (Figure 2C). Notably, PON1 in rabbit serum hydrolyzed Hcy-thiolactone (Figure 2A) even faster and afforded much better protection from protein *N*-homocysteinylation than any human serum (Figure 2C).

In a large randomized clinical trial, urinary Hcy-thiolactone was associated with myocardial infarction in coronary artery disease patients [42]. In a mouse and cellular models of Alzheimer’s disease (AD), Hcy-thiolactone promoted the accumulation of amyloid beta (Aβ) by inhibiting autophagy [15]. Hcy-thiolactone can promote the progression to AD by upregulating amyloid precursor protein (APP), which results in increased generation of Aβ [15].

The involvement of Hcy-thiolactone in disease can also be explained by its ability to impair protein structure/function via the *N*-homocystinylation of protein lysine residues [22]. For example, the *N*-homocysteinylation of fibrinogen by Hcy-thiolactone, which impairs the lysis of fibrin clots in vitro [43], explains the association of Hcy-thiolactone with the impaired lysis of fibrin clots in vivo in humans (manifested by a longer time of fibrinolysis), as we have recently shown in a large randomized controlled trial [44].

Enzymological studies in vitro led to a contention that the lactonase activity is a native physiological activity of PON1, but no physiological evidence was provided [45,46]. Other studies repeated this contention by stating that the lactonase activity is “the established native physiological activity of PONs” [47] even though no physiological evidence supporting such statement has been reported either. A study that attempted to identify endogenous lipophilic lactones as possible in vivo substrates for PON1 in human serum, found none [48]. The possible involvement of PON1 in metabolism of endogenous lipophilic lactones in vivo as proposed in refs. [45,46] remains to be proven.

Nevertheless, recent findings showed that some phenyl-γ-valerolactones (PVLs), phase 2 metabolites derived from dietary flavan-3-ols, are substrates for PON1 and PON3 in vivo [41]. Flavan-3-ols constitute the main class of polyphenolic bioactive compounds present in the food and beverages such as tea, pome fruits, cocoa products, and berries. Large-scale randomized clinical trials show that flavan-3-ol intake was associated with beneficial cardiovascular effects [49] but had no effect on cognition [50]. After intake, flavan-3-ols are catabolized by gut microbiota to PVLs and phenyl-γ-valeric acids (PVAs), which enter the circulation and are distributed throughout the human body [51]. After the intraperitoneal administration of 5-(3′,4′-dihydroxyphenyl)-*γ*-valerolactone (*γ*VL), the sulfated form of *γ*VL was detected in the brain, while *γ*VL aglycon was not detected [52]. In TNF-α stimulated human brain primary microvascular endothelial cells, 5-(4′-hydroxyphenyl)-*γ*-valerolactone-3′-sulfate and 5-(4′-hydroxyphenyl)-*γ*-valerolactone-3′-*O*-glucuronide were biologically active at low nanomolar concentrations and influenced the expression of genes involved in biological pathways such as cell adhesion, cytoskeleton organization, focal adhesion, signaling pathways, pathways regulating endothelial permeability, and interaction with immune cells [53]. However, it is not known whether corresponding PVAs (i.e., products of PVLs hydrolysis by PON1) can also influence gene expression.

**Figure 3:**
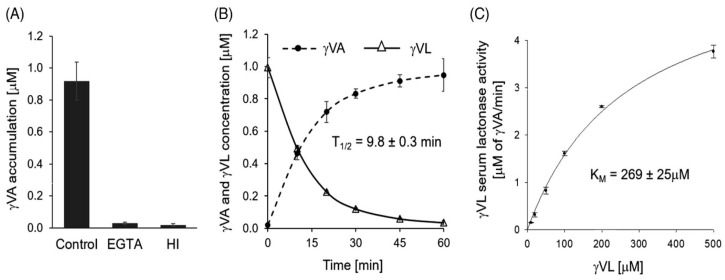
Turnover of 5-(3′,4′-dihydroxyphenyl)-γ-valerolactone (γVL) and accumulation of 5-(3′,4′-dihydroxyphenyl)-γ-valeric acid (γVA) in human serum at 37 °C. (**A**) Accumulation of γVA in control serum supplemented with 1 μM γVL after 1 h, serum + 5 mM EGTA, and heat-inactivated serum (57 °C, 30 min; HI). Data represent mean ± standard deviation (*n* = 6). (**B**) Kinetics of γVL disappearance and γVA generation in serum. (**C**) Serum γVL-hydrolyzing activity as a function of γVL concentration. Data represent mean ± standard deviation (*n* = 4 per time point or concentration). Reproduced from ref. [41].

In human serum, *γ*VL was rapidly hydrolyzed to the corresponding *γ*-substituted valeric acid (γVA) by PON1 and PON3 (t_1/2_ = 9.8 min) (Figure 3B) [41]. The hydrolysis was prevented by treatments with EGTA (calcium chelator and an inhibitor of PON1) or with heat (Figure 3A). Km was 269 μM (Figure 3C), way above sub-micromolar *γ*VL concentrations in humans [50]. Some, but not all, phase 2 metabolites (sulfated or glucuronidated γVL) were also hydrolyzed by PON1/PON3 in serum. In general, conjugated γVLs were worse substrates of PON than unconjugated γVL. Additional conjugations of γVL significantly reduced or prevented the hydrolysis of γVL metabolites by PON. Another polyphenol-derived lactone, enterolactone, was not a substrate [41].

In contrast to the established influence of the PON1 genotype on the hydrolysis of Hcy-thiolactone [23,39], the EPIC-Norfolk sub-cohort study found that the sum of urinary conjugated γVLs was not influenced by the *PON1* genotype (*Q192R*, rs662 in the coding region; −162A/G rs705381 in the promoter region) in males but there was a small effect in females [47]. These findings suggest that the *PON1* genotype has a minor sex-dependent effect on the hydrolysis of conjugated *γ*VLs; this, however, remains to be examined in future studies.

## 3. Association of PON1 with Human Disease

PON1 has been studied in the fields of toxicology, CVD, renal disease, liver disease, Alzheimer’s disease, and cancer. Initial studies have shown that *Pon1*^−/−^ mice are highly susceptible to the toxicity of organophosphate insecticides [54] and to atherosclerosis induced by the metabolic stress of a high-fat diet [55], or by *ApoE* depletion [56]. The molecular basis of PON1’s protective role in organophosphate poisoning is well understood [54]. In contrast, the nature of PON1 targets in atherosclerosis and other human diseases is not fully understood. Elevated oxidative stress and inflammation, associated with CVD [5], are also observed in Pon1-deficient mice [55,56] and humans with attenuated PON1 activity [28].

Reduced PON1 activity accompanied by increased oxidative stress and inflammation is a common finding in patients with CVD [28,30,57,58], diseases of the kidney [31,32] and liver [59], Alzheimer’s disease [33,60,61], and cancer [34,62] (Table 1). This has been suggested to be due to the mediation by PON1 of anti-oxidative and anti-inflammatory effects of HDL [35,36]. Many other HDL components have also been shown to mediate the anti-oxidative and anti-inflammatory activity of HDL [10,11,37], including APOA1, which accounts for 70% of HDL protein mass [37] and anti-apoptotic activity [38], and for most of the HDL anti-oxidative activity [8]. Although how attenuated PON1 expression or activity can induce oxidative stress and inflammation is not clear, accumulating evidence, discussed below, suggests that the influence of PON1 on oxidative stress and inflammation is indirect rather than direct [39].

### 3.1. PON1, Oxidative Stress, Inflammation, and CVD

In *Pon1*^−/−^ mice fed with a high-fat diet, the enhancement of atherosclerosis is accompanied by the upregulation of oxidative stress, which is manifested by elevated levels of lipid peroxides in purified HDL [55]. In *Pon1*^−/−^*ApoE*^−/−^ mice, elevated oxidized phospholipid epitopes in plasma, bioactive oxidized phospholipids in purified endogenous intermediate density lipoprotein/LDL, and the upregulated expression of oxidative stress-responsive genes such as heme oxygenase-1 (HO1), peroxisome proliferator-activated receptor gamma (PPARγ), and oxLDL receptor (oxLDL-R) in the liver were observed [56]. Although no lipid hydroperoxides were found in fresh purified LDL from any Pon1 genotype, the LDL from *Pon1*^−/−^ mice nevertheless stimulated lipid hydroperoxide generation and monocyte transmigration better than did the LDL from *Pon1*^+/+^ mice in a coculture model. These results suggested that the LDL from *Pon1*^−/−^ mice was changed somehow to become prone to oxidation. Lipid hydroperoxide formation in LDL was inhibited by the pretreatment with purified human PON1 [55].

The overexpression in mice of human PON1 using bacterial artificial chromosome genomic clones increased plasma PON1 levels 2- to 4- fold and significantly reduced aortic lesions in dietary as well as *ApoE*^−/−^ models [64]. The overexpression of human PON1 in a *LDL*^−/−^ mouse model of metabolic syndrome via adenovirus-mediated PON1 gene transfer increased the paraoxonase activity of PON1 4.4-fold and significantly reduced the plaque-associated oxLDL, titer of auto-antibodies against LDL modified by malondialdehyde (MDA) (a proxy for oxLDL), and plaque volume by 80% [65]. Unfortunately, how Pon1 overexpression influences the expression of genes involved in inflammation and oxidative stress has not been studied in these two mouse models.

The overexpression of the human PON gene cluster (PC Tg) in *ApoE*^−/−^ mice using bacterial artificial chromosome increased the enzymatic activity towards the paraoxon substrate in isolated HDL by 60%, stabilized atherosclerotic lesions, and significantly attenuated (by 20–30%) plaque area [66]. PC Tg HDL significantly inhibited oxLDL production (by 40–67%) compared to wild-type HDL. The inflammation markers ICAM-1 and MCP-1 were significantly downregulated (by 31–51%) in PC Tg/*ApoE*^−/−^ mice compared to *ApoE*^−/−^ mice. The inflammatory response of isolated PC Tg macrophages (due to human PON2 overexpression) was significantly attenuated compared to macrophages isolated from *ApoE*^−/−^ mice, as shown by the quantification of TNF-α and IL-6. Human PON2 inhibited also macrophage MMP-9 expression and foam cell formation from PC Tg macrophages compared to macrophages from *ApoE*^−/−^ mice [66]. These findings show that PON gene cluster overexpression protects from atherosclerosis by ameliorating oxidative stress and downregulating the expression of genes involved in inflammation.

The first large-scale prospective study that evaluated the relationship between oxidative stress and the PON1 genotype, and their activity and prognostic value as predictors of future CVD, involved 1339 patients (65 years old, 72% male) and 283 controls without CVD (57 years old, 48% male) who underwent coronary angiography [28]. The study found, at baseline, that the low paraoxonase or arylesterase activity of PON1 as well as the *PON1-192QQ* polymorphic variant were associated with elevated various oxidized fatty acids (5-, 8-, 9-, 11-, 12-, 15-hydroxyeicosatetraenoic acids (HETEs), 9-, 13-hydroxyoctadecadienoic acids (HODEs), 8-isoprostane prostaglandin F_2α_ (8-isoPGF_2α_)) in patients (*n* = 150) (Table 1). Participants carrying *PON1-192QQ* alleles had a significantly increased risk of adverse CVD outcomes such as death, myocardial infarction (MI), and stroke, compared with *PON1-192RR* and *PON1-192QR* carriers over a 3-year-followup (18.0% vs. 13.6%, adjusted hazard ratio 1.48, *p* = 0.01) and all-cause mortality (11.1% vs. 6.75%, adjusted hazard ratio 2.05, *p* = 0.001). Although the *PON1-192QQ* genotype was not associated with nonfatal stroke or MI, the low activity of PON1predicated an increased frequency of stroke and MI, all-cause mortality, and the sum of adverse CVD outcomes. Specifically, participants with the lowest paraoxonase or arylesterase activity of PON1 (1st quartile) showed an increased frequency of adverse CVD outcomes (paraoxonase: 25.1%; arylesterase 23.5%) compared to participants with the highest activities of PON1 (4th quartile) (7.3% or 7.7%, respectively). The adjusted hazard ratios for nonfatal MI and stroke, all-cause mortality, and the sum of in the lowest vs. highest PON1 activity quartiles were 4.4, 2.4, and 3.4, respectively, for paraoxonase and 4.5, 2.2, and 2.9 for arylesterase, respectively, they were independent in multivariate analysis in models (separate for paraoxonase and arylesterase) adjusted for all traditional cardiac risk factors and medications). These findings demonstrate that the PON1 genotype/activity affects oxidative stress and predicts future CVD risk.

However, it should be noted that the *PON1-Q192R* polymorphism, associated with indices of oxidative stress in vivo [28], has opposite effects on the paraoxonase and arylesterase activities of PON1: The *192Q* allele associates with low paraoxonase and high arylesterase activity, while the *192R* allele associates with high paraoxonase and low arylesterase activities [23,26,29,67]. In this context, it is not clear why the low paraoxonase and arylesterase activities of PON1 were associated with nonfatal stroke and MI, but the *PON1-192QQ* genotype was not [28].

A population-based cross-sectional study of 1,895 participants (32-year-old, 46% male) examined a relationship between rs669 SNP (*PON1-Q192R*), PON1 activity, and conjugated dienes in lipoprotein lipids [68] as a measure of oxLDL lipids [69] that is known to correlate with lipid hydroperoxides and MDA [70]. In multiple regression models, the paraoxonase activity of PON1was inversely correlated with oxLDL lipids (*p* = 0.0001) but not with oxHDL lipids, and tended to be associated with oxLDL protein (*p* = 0.08). A stronger association between PON1 activity and oxLDL lipids was seen in the *PON1-192RR* carriers than in the *PON1-192QQ* carriers. Although *PON1 rs662* SNP was strongly associated with paraoxonase activity of PON1, it was not associated with oxHDL lipids or protein.

A case-control study found that CAD patients confirmed by angiography (*n* = 105) had significantly increased plasma 8-isoprostane F2 (8-iso-PGF2α, produced by the non-enzymatic peroxidation of arachidonic acid in membrane phospholipids) and reduced paraoxonase and arylesterase activities of PON1 compared to healthy controls (*n* = 45) [58]. Paraoxonase and arylesterase activities of PON1 were significantly negatively associated with the severity of CAD (Gensini score) in univariate analyses, while 8-iso-PGF2α was associated positively. Such associations were also seen in multiple regression models adjusted for traditional risk factors. These findings suggest that PON1 may protect phospholipids from oxidation [58]. However, the mechanism underlying these findings remains to be elucidated.

One study examined how *PON1* SNPs and PON1 arylesterase activity are related to the oxidation susceptibility of LDL isolated from male CAD patients and healthy control participants [71]. The susceptibility of LDL to oxidation was measured using an assay, in which LDL is oxidized by copper and the resulting conjugated dienes are monitored by absorbance at 234 nm. During LDL preparation from each participant, HDL/PON1 was removed. The study involved CAD patients (*n* = 205, 70-year-old, > 80% stenosis) and control participants (*n* = 232, 66 years old, <15% stenosis). It was found that the susceptibility of LDL to oxidation was not correlated with arylesterase activity of PON1, although it was correlated with CAD. In contrast, PON1 promoter SNP, *PON1 -108C/T*, which affects PON1 expression, and other *PON1* SNPs, were associated with the susceptibility of LDL to copper induced. The absence of congruency in the relationships between the susceptibility of LDL to oxidation and CAD, PON1 arylesterase activity, and *PON1 -108C/T* SNP raises doubts regarding validity of the experimental approach used in this study.

### 3.2. PON1, Lipid Oxidation, Hcy-Thiolactone, and Alzheimer’s Disease

OxLDL lipids increase β-amyloid production by SH-SY5Y cells [72]. Importantly, amyloid beta binds to oxLDL and accelerates the formation of macrophage foam cells [73], suggesting that oxLDL can directly participate in the development of AD.

**Figure 4:**
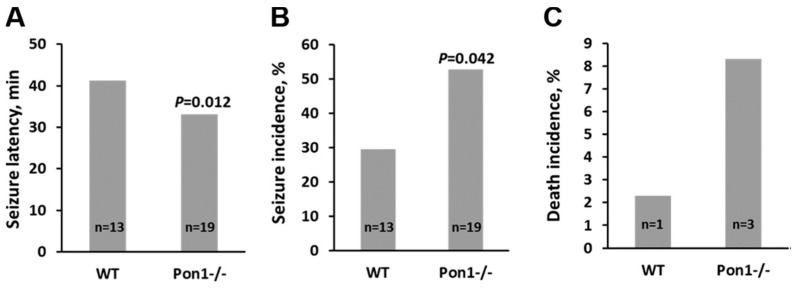
Latency (**A**), incidence of seizures (**B**), and death (**C**) in *L*-Hcy-thiolactone-injected (intraperitoneally, 3.7 μmol/g body weight) *Pon1*^−/−^ mice (*n* = 19) and their *Pon1*^+/+^ siblings (WT, *n* = 13) monitored for 90 min after injection. The figure was drawn based on data from Borowczyk et al. [40].

PON1 plays an important role in the detoxification of neurotoxins including organophosphate pesticides, potent inhibitors of acetylcholinesterase; people with low PON1 activity show increased sensitivity to neurotoxins while those with high PON1 activity are less susceptible [55]. Notably, exposure to organophosphates increases the risk of developing AD [74,75]. Treatments with doses of chlorpyrifos oxone that do not affect wild-type *Pon1*^+/+^ mice are known to induce seizures and death in *Pon1* knockout mice [55]. Importantly, Pon1 is also known to detoxify Hcy-thiolactone in mice. For example, treatments with Hcy-thiolactone induce seizures significantly faster (Figure 4A) and with increased incidence (Figure 4B) in *Pon1*^−/−^ mice compared to wild type *Pon1*^+/+^ mice. Death incidence was also higher in *Pon1*^−/−^ mice (Figure 4C) [40].

#### 3.2.1. Mice

Proteomic analyzes of *Pon1*^−/−^ and *Pon1*^+/+^ mice have shown that Pon1 plays an important role in maintaining cellular proteostasis [60], in addition to controlling Hcy-thiolactone and *N*-Hcy-protein levels [40]. *Pon1* gene deletion affects the expression of cellular proteins in an organ-specific way, with the patterns of expression modulated by hyperhomocysteinemia (HHcy). In the brains of *Pon1*^−/−^ mice, proteins involved in anti-oxidant defenses (Sod1, DJ-1), brain-specific function (Nrgn), and the assembly of cytoskeleton (Tbcb) were significantly downregulated, while the CapZa2 protein involved in the assembly of cytoskeleton was significantly upregulated compared to wild-type *Pon1*^+/+^ mouse brain [60].

In the brains of HHcy *Pon1*^−/−^ mice that were fed with high methionine diet, Prdx2 and DJ-1proteins participating in anti-oxidant defense; Ncald, Nrgn, and Stmn1 proteins involved in brain-specific function; energy metabolism protein Ak1; cell cycle GDI1 and Ran proteins; cytoskeleton assembly Tbcb protein; and Hdhd2 protein of unknown function were all upregulated (Table 2). Notably, *Pon1* gene deletion affected the expression of DJ-1 (Park7), Sod1, and Prdx2 proteins involved in the oxidative stress response that are also known to be associated with AD [60].

Clusterin (CLU or APOJ), involved in the transport of amyloid beta (Aβ) from plasma to the brain in humans (reviewed in [76]), is carried on a minor HDL subspecies that contains two other proteins: APOA1 and PON1 [77]. Importantly, Clu (ApoJ) levels are significantly elevated in the plasma of *Pon1*^−/−^ mice compared to wild-type *Pon1*^+/+^ animals [23].

PON1 involvement in AD was examined using *Pon1*^−/−^5xFAD mice and in Aβ-overexpressing mouse neuroblastoma N2a-APP_swe_ cells [15]. 5xFAD mice overexpress the K670N/M671L (Swedish), I716V (Florida), and V717I (London) mutations in human APP (695) and the M146L and L286V mutations in human PS1 and start to accumulate high levels of Aβ42 at about 2 months old [78]. The dysregulation of mTOR signaling and autophagy are linked to Aβ accumulation in AD patients [79,80], while histone H4K20me1 demethylation by the histone demethylase PHF8 maintains the homeostasis of mTOR signaling [81].

**Figure 5:**
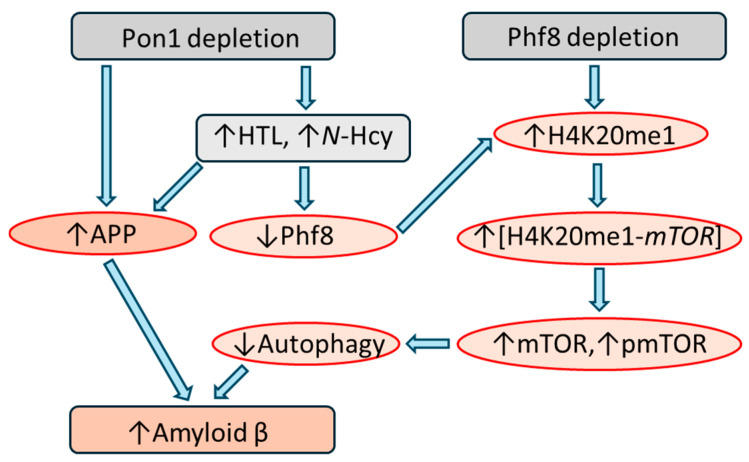
Proposed mechanism of Aβ generation in *Pon1*^−/−^5xFAD mice. Pon1, paraoxonase 1; Hcy, homocysteine; HTL, Hcy-thiolactone; APP, amyloid β precursor protein; mTOR, mammalian target of rapamycin; pmTOR, phospho-mTOR; Phf8, plant homeodomain finger protein 8. [H4K20me1-*mTOR*] represents H4K20me1 bound at the *mTOR* promoter. The thick arrows indicate the influence of the change in one process/protein/metabolite on another. The thin up and down arrows indicate the direction of change. Reprinted from ref. [83].

The study revealed that Pon1 plays an important role in protecting from the amyloidogenic processing of APP to Aβ in brains of mice and identified mechanism of this new function of Pon1 in the central nervous system (Figure 5). Specifically, Pon1 depletion in *Pon1*^−/−^5xFAD mice significantly downregulated Phf8 and upregulated the methylated histone H4K20me1 mark. This led to the upregulation of mTOR expression and increased its active form, phospho-mTOR, which impaired autophagy by downregulating Bcln1, Atg5, and Atg7 proteins in *Pon1*^−/−^5xFAD mouse brains compared to *Pon1*^+/+^5xFAD brains. Silencing of the *Pon1* gene in N2a-APP_swe_ cells by RNA interference using the siRNA targeting *Pon1* gene led to Phf8 downregulation, which increased histone H4K20me1 binding at the *mTOR* promoter, thereby upregulating mTOR expression and signaling. Upregulated mTOR signaling impaired autophagy and significantly elevated APP expression and Aβ levels. Hcy-thiolactone or *N*-Hcy-protein (metabolites known to accumulated *Pon1*^−/−^ mice), or Phf8 depletion by RNA interference, elevated Aβ levels in N2a-APP_swe_ cells [15]. Notably, *Phf8* gene silencing did not influence the expression of APP, indicating that Aβ levels increased independently of APP [82].

Pon1 depletion induced changes in the Phf8->H4K20me1->mTOR->autophagy pathway (Figure 5) that were similar to the changes induced by HHcy [15], suggesting that the same Hcy metabolites were involved. This suggestion is supported by our previous findings showing that a common primary physiological outcome of Pon1 depletion and of HHcy was essentially the same: Pon1 depletion [40,84] and HHcy [85] each increased Hcy-thiolactone and *N*-Hcy-protein levels. Taken together, our findings show that Aβ generation in the *Pon1*^−/−^ brain is mediated by the influence of Hcy-related metabolites on mTOR signaling/autophagy [15]. These findings provide a mechanistic explanation for the link between attenuated PON1 activity [36] or elevated Hcy [86] and AD.

#### 3.2.2. Humans

A few studies have examined PON1 activity in relation to oxidative stress in AD patients. In one study, PON1 activities were found to be significantly decreased (Table 1) while PAF-AH activity and oxLDL levels were significantly increased in 49 AD patients (74 years old, 59% female, MMSE score = 21 ± 5) compared to 34 age/sex-matched control individuals [63]. The study found a significant inverse correlation of oxLDL with PON1 activities (but not with activity of PAF-AH) in AD patients and non-AD control individuals. Further, the activities of PON1 and levels of oxLDL were associated with the severity of AD (assessed by using the MMSE test, which quantifies global cognition). Patients with moderate (MMSE score of 11 to 24) and severe (MMSE score < 10) AD had significantly decreased activities of PON1 and increased oxLDL levels compared to patients with mild AD (MMSE score > 24). Even though PAF and oxidized phospholipids hydrolysis by PAF-AH generates free oxidized fatty acids, which have potent biological activity, PAF-AH activity was not correlated with the oxLDL nor with the severity of AD. Although these findings suggest that PON1 may participate in oxLDL metabolism in AD, the nature of this participation is not clear.

Another study evaluated oxLDL in 54 late-onset AD patients (aged 77 years, 81% female, MMSE score = 18) and 51 healthy elderly individuals (aged 77 years, 73% male, MMSE score = 29) and a relationship between oxLDL and *PON1-107C/T* polymorphism and the *APOE* genotype [87]. Patients with AD and control individuals with the *PON1-107TT* genotype had significantly elevated levels of plasma oxLDL compared to those with the *PON1-107CC/CT* genotype. The distribution of lipoprotein cholesterol in patients with AD was shifted toward a greater prevalence of smaller, denser LDL. In AD patients, the smaller, denser LDL levels were significantly associated with the levels of oxLDL. Lipoprotein distribution was not influenced by *APOE* genotype. These findings suggest that plasma oxLDL levels could modulate the association of *PON1-107TT* polymorphism with AD [87].

A study that examined relationships among PON1, lipid peroxidation, and dementia with AD patients (*n* = 63), vascular dementia patients (*n* = 40), and mixed dementia patients (*n* = 33) found that MDA/thiobarbituric acid-reactive substances were elevated to a greater extent in vascular dementia than in AD [61]. In patients with vascular involvement, the increase in MDA/TBARS reflected the extent of global cortical atrophy. The arylesterase activity of PON1 was significantly attenuated in patients with dementia, more so in patients with severe cognitive deficits. In patients with vascular dementia, the low arylesterase activity of PON1 was associated with increased brain ischemia and medial temporal lobe atrophy. These findings show that the reduced activity of PON1 and increased levels of the MDA/TBARS oxidative stress marker are associated with brain atrophy and vascular dementia rather than with cognitive decline. However, it is not clear how reduced PON1 activity can lead to oxidative stress and impaired cognition.

### 3.3. PON1 Depletion, Dysregulation of Signaling Pathways, and Cancer

Hepatocellular carcinoma (HCC) is one of the most common neoplasms, the third leading cause of cancer death, and a leading cause of death among patients with cirrhosis [88]. Liver cirrhosis is widely prevalent worldwide and can be a consequence of different causes, such as obesity, non-alcoholic fatty liver disease, high alcohol consumption, hepatitis B or C infection, autoimmune diseases, cholestatic diseases, and iron or copper overload [59].

Recent studies show that PON1 activity and expression are compromised in HCC patients. Specifically, serum PON1 activity, measured with 4-nitrophenylacetate as a substrate, was significantly reduced in HCC patients [62]. Transcriptomic analysis showed that the expression of PON1was significantly downregulated in HCC tissues compared to normal tissues [34]. However, there was also a significant variation in PON1 expression between HCC patients. Patients with low PON1 expression manifested significant differences in pathology severity and tumor size and grade. Female HCC patients with low PON1 expression had a higher degree of tumor malignancy.

Differences in PON1 expression influenced the clinical manifestations, biological processes, immune infiltration, and expression of immune checkpoints in HCC, suggesting that PON1 plays an important role in modulating tumor progression and immune cell infiltration, thus establishing PON1 as a new biomarker important for prognosis, targeted therapy, and immunotherapy in HCC patients [34].

Bioinformatic analysis of pathway enrichment in the high and low PON1 mRNA expression groups showed that the *PON1* gene inhibits key signaling pathways, such as the PI3K/Akt/mTOR signaling, the cell cycle G2 checkpoint, the TGF-β signaling, and the Wnt/β-catenin signaling, which play a crucial role in pathogenesis and progression of HCC [34]. Interestingly, we found that Pon1 depletion upregulated mTOR signaling and inhibited autophagy via Pcft/H4K20me1 in mouse brain and neuroblastoma cells [15], suggesting that effects of Pon1 depletion on gene expression are disease/tissue-specific.

## 4. PON1 Has No Intrinsic Anti-Oxidant Activity: Don’t Waste Clean Thoughts on Dirty Enzymes

PON1 has been stated to hydrolyze oxidized lipids and, thus, to promote atheroprotective effects, e.g., refs. [28,89], which incorrectly implies that PON1 has an intrinsic anti-oxidant function. That PON1 has the ability to hydrolyze oxidized lipids was originally proposed by a study that reported the ability of purified native human PON1 to inhibit copper-induced oxidation of LDL in an in vitro assay that quantified lipo-peroxides and TBARS [90].

The availability of an assay for a biological event in a cell-free system usually facilitates studies of its molecular mechanism. Indeed, this assay has been used in many in vitro studies using purified native (e.g., refs. [91,92,93,94]) and recombinant [95,96] PON1 preparations. Unfortunately, these and other studies in the PON1 field did not follow the maxim “don’t waste clean thoughts on dirty enzymes” attributed by Arthur Kornberg in his ‘ten commandments of enzymology’ [97] to Efraim Racker, a pioneer in the enzymology of oxidative phosphorylation [98].

Some labs did not replicate the finding that purified PON1 protects LDL from oxidation [46,99] while those that did [100,101,102] went on to correct themselves by showing, in more rigorous and well-controlled studies, that their earlier findings were due to PAF-AH contamination in PON1 preparations and that PAF-AH-free PON1 does not protect lipoproteins from oxidation nor hydrolyze oxidized lipids [46,103,104]. Rigorously purified PON1, or plasma from an individual with a mutation in the *PAF-AH* gene, did not hydrolyze PAF nor the oxidized phospholipids from oxLDL [99].

One study purported to show that purified PON1 was capable of hydrolyzing PAF. In that study [105], purified PON1 preparations were tested by Western blotting (20 μg) and amino acid sequencing (50 μg, or about 1 nmol PON1) and found not to have any detectable PAF-AH contamination. However, such evidence does not exclude PAF-AH contamination, considering that as little as 5 to 10 ng of PAF-AH (undetectable by Western blotting and not sufficient for sequencing) is sufficient to account for all the phospholipase activity in purified PON1 preparations [99]. In fact, other labs have shown that purified PON1 has no phospholipase A2-like activity toward PAF or pro-atherogenic oxidized phospholipids and that PAF-AH is the sole phospholipase A2 of HDL [99,103]. Rigorously purified PON1, or plasma from an individual with a mutation in the *PAF-AH* gene, did not hydrolyze PAF nor the oxidized phospholipids from oxLDL [99].

Although Aviram et al. [95] and Liu et al. [96] reported that PON1, PON2, and PON3 protect LDL from oxidative modification, Draganov et al. found no protection [46]. Re6combinant human PON1 was expressed from a baculovirus vector in insect cells and purified. When PON1 hydrolytic activity and a putative anti-oxidant activity were monitored during PON1 purification, the two activities did not co-purify at any stage and in any of the preparations. The putative anti-oxidant activity was shown to be associated with a low mass contaminant and the detergent used in PON1 purification [46]. That putative anti-oxidant activity in PON1 preparations was associated with the detergent present in these preparations was confirmed by another lab that also showed that anti-oxidant activity was not associated with hydrolytic PON1 activities such as arylesterase and lactonase, nor with phospholipase activity [103]. Unfortunately, it appears that many other laboratories did not seem bothered to control their PON1 preparations for contaminants.

Studies that examined the contribution of individual protein components to the ability of HDL to inhibit LDL oxidation showed that APOA1 is the major anti-oxidant protein in HDL [10]. APOA1 is also the major factor responsible for the protection of human endothelial cells from oxLDL-induced apoptosis, accounting for 70% of HDL antiapoptotic activity [37]. APOA1 is one of the two phosphatidylcholine peroxide-reducing enzymes isolated from human plasma (the other is glutathione peroxidase) [106]. APOA1 is essential for HDL structure and for activation of the HDL-associated enzymes PON1 and LCAT [107]. Two methionine residues in APOA1 (Met112, Met148) are oxidized to sulfoxides during the reduction of lipid peroxides to redox-inactive hydroxides [108,109]. Reconstituted HDL containing only purified APOA1 and phospholipids (palmitoyloleoyl phosphatidylcholine at a molar ratio of 1.0/77.1) has the capacity to inhibit LDL oxidation like that of native normolipidemic small, dense HDL3b and 3c isolated from normal human plasma. The oxidation of APOA1 Met residues in HDL3 incubated with oxLDL is accompanied by the concomitant reduction of lipid peroxides to lipid hydroxides [8].

To assess a role of HDL-associated enzymes, such as PON1, PAF-AH, and LCAT, in oxLDL inactivation, HDL3 was pretreated with inhibitors such as DFP, which inhibits the 3 enzymes, Pefabloc, which inhibits only PAF-AH, or EDTA, which inhibits only PON1, and then incubated with oxLDL. As expected, pretreatment significantly reduced the activities of LCAT (by 50%), PAF-AH (by 90%), and PON1 (by 99%). In contrast, the capacity of HDL3 to inactivate lipid peroxides in oxLDL or to delay LDL oxidation was not affected. None of the inhibitors impaired the capacity of HDL3 to delay the accumulation of conjugated dienes in LDL [8]. Two earlier studies have also reported that the inactivation of PON1 activity by EDTA did not affect the anti-oxidant activity of HDL3 [110] or PON1 preparations [93] in the copper-induced LDL oxidation assay. These findings do not support the contention that paraoxonase activity inhibits the formation of ‘minimally oxidized’ LDL by hydrolyzing biologically active oxidized phospholipids [9,91].

## 5. Mechanistic Bases of PON1 Involvement in Human Disease

The findings that PON1 is associated with oxidative stress and inflammation in humans and in mouse models (discussed in Section 2) but cannot be directly linked to these processes (discussed in Section 3), suggest that the influence of PON1 on oxidative stress and inflammation is indirect.

Studies of proteomic and transcriptomic changes in mice and humans in relation to changes in PON1 expression and activity (listed in Table 3 and discussed below) support this suggestion and provide insights into the biological function of PON1. Specifically, these studies suggest that vascular inflammation and oxidative stress that are associated with PON1 depletion are caused by the dysregulation of genes involved in these processes. The majority of studies discussed in this section are related to CVD, with two related to kidney disease and one to brain disease. Studies related to AD and cancer are discussed in Section 2.

### 5.1. Low PON1 Activity in Dysfunctional HDL Is Associated with Impaired Nitric Oxide Production in Endothelial Cells

Native HDL possesses anti-inflammatory and anti-oxidative properties [35] and directly influences the vascular endothelium by the activation of nitric oxide (NO) synthesis by eNOS [118,119], thus promoting endothelial repair [120]. Such cardio-protective processes are, at least in part, mediated by the binding of HDL to endothelial scavenger receptor B, type I (SR-BI), and by PON1 [6], carried in the circulation in a minor fraction of HDL [16].

Patients with stable coronary artery disease (CAD) or an acute coronary syndrome carry dysfunctional HDL_CAD_, which does not promote the endothelial NO synthesis, anti-inflammatory effects, and repair that are characteristic of normal HDL isolated from healthy individuals [6]. This has been shown to be due to activation by HDL_CAD_ of the endothelial lectin-like oxLDL receptor 1 (LOX-1), which induces endothelial PKCβII activation, thereby inhibiting eNOS-dependent NO generation. These newly acquired properties were conferred by elevated MDA, a product of lipid peroxidation, which chemically modified PON1, thereby reducing its activity and generating dysfunctional HDL_CAD,_ which activates PKCβII and lacks the anti-oxidative, anti-inflammatory properties of normal HDL. Moreover, HDL from *Pon1*^−/−^ mice failed to enhance endothelial NO production, while the addition of pure PON1 or HDL from healthy individuals partially ameliorated the stimulating effects of HDL on NO production. Even though PON1 activity in HDL_CAD_ was decreased, PON1 protein content in HDL_CAD_ was elevated compared to HDL from healthy controls, suggesting that the enzymatic activity of PON1 was inactivated in HDL_CAD_. These findings show that HDL-associated PON1 activity has an important function in maintaining the ability to stimulate endothelial-atheroprotective effects of HDL, *i.e.*, NO production. The impairment of this fundamental role of PON1/HDL by oxidative stress can account for the increased risk of adverse cardiovascular events in CAD patients [28,29]. These findings also show that PON1 regulates the expression of genes involved in endothelial homeostasis and that the dysregulation of these processes leads to CAD or acute coronary syndrome.

### 5.2. Pon1 Depletion Affects Expression of Genes Involved in Inflammation, Oxidative Stress, and Blood Clotting

Although Pon1 depletion in mice in the absence of hyperlipidemia does not induce atherosclerosis [55], *Pon1*^−/−^ mice fed with a standard normolipidemic chow diet show an altered expression of proteins involved in vascular inflammation, oxidative stress, and thrombogenicity [121]. Specifically, there was a significant 2-fold increase in leukocyte adhesion revealed by intravital microscopy, but no significant change in leukocyte rolling in *Pon1*^−/−^ mice compared to *Pon1*^+/+^ control animals. The increase in adhesion was correlated with significant increases in aortic *P-selectin* and *Icam* mRNA levels (*p* = 0.016) and a 1.3-fold increase in *Vcam1* mRNA (*p* =0.096). Aortic Tnf-α mRNA expression was not affected. The rate of aortic superoxide production was significantly increased in *Pon1*^−/−^ vs. *Pon1*^+/+^ mice (3-fold, *p* = 0.04). *Pon1*^−/−^ mice were also predisposed to thrombosis, as shown by a significant 57% reduction in time to occlusion in a carotid thrombosis assay (*p* < 0.001). Notably, these vascular changes mimic those seen in severely hyperlipidemic *ApoE*^−/−^ mice [121]. These findings also show that *Pon1* interacts with genes involved in inflammation, oxidative stress, and blood clotting.

### 5.3. Pon1 Depletion Increases Expression of Liver Oxidative Stress Genes and Accelerates Atherosclerosis

In mice fed with a high-fat diet, *Pon1* gene deletion led to increased atherosclerosis and increased lipid peroxides levels in isolated HDL compared to *Pon*^+/+^ animals [55]. In *ApoE*^−/−^ mice, *Pon1* gene deletion also increased atherosclerosis and oxidative stress, manifested by elevated epitopes in plasma-oxidized phospholipid, biologically active oxidized phospholipids in isolated endogenous intermediate-density lipoprotein/LDL. RT-qPCR analyses showed that these changes were accompanied by the upregulated expression of genes involved in oxidative stress-responsive genes in the liver) HO-1, PPARγ, and oxidized LDL-R) [56] (Table 3). These findings show that Pon1 deficiency promotes oxidative stress and atherogenesis. These results also suggest that Pon1 interacts with oxidative stress-responsive genes and that the disruption of these interactions induces oxidative stress and causes atherosclerosis.

### 5.4. Pon1 Depletion Increases Expression of Oxidative Stress Genes in Liver, Kidney, and Brain

Proteomic analyses of *Pon1^−/−^* mice show that, in addition to controlling Hcy-thiolactone and *N*-Hcy-protein levels [40], Pon1 is important in maintaining cellular proteostasis. Specifically, in the brains of *Pon1^−/−^* mice fed with a normal chow diet, the levels of proteins involved in anti-oxidant defenses (Sod1, DJ-1) were significantly reduced compared to *Pon*^+/+^ animals. In the presence of hyperhomocysteinemia (HHcy) induced by feeding with a high-methionine diet, DJ-1 and Prdx2 proteins were significantly upregulated in HHcy *Pon1^−/−^* mice compared to HHcy *Pon*^+/+^ animals [60]. In the kidneys [113] and livers [112] of *Pon1^−/−^* mice, the levels of the anti-oxidant protein Prdx2 were significantly elevated compared to *Pon*^+/+^ animals. These findings suggest that Pon1 interacts with oxidative stress-responsive proteins in an organ-specific way and that HHcy modulates these interactions.

### 5.5. Pon1 Depletion in Scarb1^−/−^ Mice Is Associated with Upregulated Expression of Oxidative Stress Genes

Scavenger receptor BI (SR-BI) plays a central role in reverse cholesterol transport (RCT) as the major receptor for HDL cholesterol (HDL-C) [122]. Although elevated plasma HDL-C levels are associated with a lower risk of CVD in humans, a rate mutation in the human *SCARB1* gene encoding SR-BI increases the risk of CVD, suggesting that high concentrations of HDL-C are not causally protective against CVD and that cholesterol flux and HDL function are more important than the steady-state levels [123]. The SR-BI knockout mice (*Scarb1*^−/−^ mice) have dysfunctional HDL characterized by impaired macrophage reverse cholesterol transport (RCT) [124], high plasma HDL-C levels, and impaired anti-oxidative and anti-inflammatory properties, and are susceptible to atherosclerosis [125].

Notably, the dysfunctional HDL is also characterized by reduced PON1 arylesterase and paraoxonase activities and is associated in a tissue-dependent way with indices of oxidative stress such as isoprostane F2α-VI (iPF2α-VI) and protein carbonyls in *Scarb1*^−/−^ mice. The levels of monocyte chemoattractant protein-1 (MCP1) were similar in *Scarb1*^−/−^ and wild-type mice, indicating that SR-BI deletion has no effect on inflammation. A Western diet did not affect MCP1 levels in *Scarb1*^−/−^ mice but increased serum PON1 paraoxonase activity and urinary iPF2α-VI in both *Scarb1*^−/−^ and wild-type mice [126].

The dysfunctional HDL and reduced PON1 activity in *Scarb1*^−/−^ mice were associated with the upregulated expression of genes encoding oxidative stress proteins. Specifically, mRNAs for glutathione peroxidases GPx1 and GPx4, superoxide dismutase SOD1 and SOD2, glutathione S-transferases GSTA2 and GSTA4, which reduce lipid peroxidation products, and HO-1, which removes free prooxidant heme and generates of the anti-oxidant bilirubin, were upregulated in *Scarb1*^−/−^ mice compared to wild-type animals. PAF-AH activity and catalase expression were not affected by *Scarb1* depletion and reduced Pon1 activity [126].

### 5.6. Pon1 Depletion in Scarb1^−/−^ Mice Affects Expression of Oxidative Stress and Inflammation-Related Liver Genes

Lipo-proteomics analysis showed that the protein content of dysfunctional HDL from *SR-BI*^−/−^ mice was decreased by 25%, compared to wild-type *SR-BI*^+/+^ animals [115]. Out of 78 proteins identified in *SR-BI*^−/−^ HDL, 26 were upregulated and 10 were downregulated compared to *SR-BI*^+/+^ HDL. Specifically, ApoA1, ApoA2, ApoC1, ApoC2, ApoM, and PON1 were downregulated, while ApoE, ApoH, Lcat, acute phase proteins ApoA4, Saa, complement C3, proteinase inhibitors such as A1AT, inter alpha-trypsin inhibitor (3 of Itih1-4), and α-2-macroglobulin were upregulated in *SR-BI*^−/−^ HDL compared to *SR-BI*^+/+^ HDL. Interestingly, plasma proteomics showed that these proteins were also affected in the plasma of *Pon1*^−/−^ mice compared to wild-type *Pon1*^+/+^ animals (Table 3) and in *PON1-Q192R* polymorphism in humans [23]. Proteins involved in lipid metabolism were significantly decreased (37.34% vs. 57.98%), as were anti-oxidant proteins (1.94% vs. 7.09%). In contrast, proteins involved in inflammatory/immune response were significantly increased (22.14% vs. 9.19%), as were proteinase-inhibiting proteins (15.67% vs. 8.49%). In in vitro experiments, *SR-BI*^+/+^ HDL significantly reduced Mcp1 and Tnf-α levels in oxLDL-treated macrophages while *SR-BI*^−/−^ HDL had no effect [115].

Probucol is a cholesterol-lowering and anti-oxidant drug [127] that rescues female infertility in *SR-BI*^−/−^ mice [128]. Treatments with probucol lowered plasma total and free cholesterol mainly in the HDL-C fraction, upregulated Pon1 and ApoA1, and downregulated ApoA4, Saa, A1AT, and myeloperoxidase (MPO) activity in *SR-BI*^−/−^mice. These findings indicate that the anti-oxidant properties of HDL were improved by the probucol treatment [115].

### 5.7. PON1 Regulates the Expression of Hepatic Genes Involved in HDL Metabolism, Oxidative Stress, and Inflammation

Lentivirus-mediated Pon1 overexpression resulted in a significant elevation of PON1 levels in liver and plasma by 63% in *SR-BI*^−/−^ mice [117]. Pon 1 overexpression improved the anti-oxidative and anti-inflammatory properties of dysfunctional HDL and reduced hepatic steatosis and aortic atherosclerosis through its effects on the expression of genes involved in these processes. Specifically, cholesterol transporter *Scarb1*, inflammatory cytokines *Il-6*, *Tnf-α*, and *Nox1* mRNAs were significantly downregulated, while *Abca1* mRNA and the anti-inflammatory cytokines *Il-4* and *Il-10* were upregulated in macrophages treated with plasma from Pon1^+^*SR-BI*^−/−^ mice. The levels of plasma MPO activity, an oxidative enzyme secreted by activated neutrophils, monocytes, and macrophages, were significantly reduced in *Pon1*^+^*SR-BI*^−/−^ mice. Lecithin-cholesterol acyltransferase (Lcat) (which removes cholesterol from the blood and tissues), ApoA1, and ApoE were significantly upregulated in *Pon1*^+^*SR-BI*^−/−^ plasma. Histological examinations showed that aortic lesions and hepatic lipid depositions were significantly reduced in *Pon1*^+^*SR-BI*^−/−^ mice. Hepatic ApoA1, ApoE, LDLR, LXRα, Abca1, Abcg5/8, and Acat were significantly upregulated, while inflammatory cytokines Il-6 and Tnf-α were downregulated, in *Pon1*^+^*SR-BI*^−/−^ mice. These findings indicate that Pon1 can regulate proteins important for HDL function and ameliorate atherosclerosis and hepatosteatosis [117].

### 5.8. Pon1 1 Ameliorates Renal Lipotoxicity by Regulating Genes Involved in Activating Lipophagy and Inhibiting Pyroptosis

Excessive lipid accumulation can lead to lipotoxicity due to the generation of toxic lipid intermediates. In the kidney, one of the more vulnerable organs, lipotoxicity causes tissue damage and dysfunction via oxidative stress, inflammation, and autophagy impairment, which lead to renal disease [129]. Pon1 is expressed in the glomerular clusters and the epithelial cells of the proximal tubules in the kidney [130]. *Pon1^−/−^* mice show increased oxidative stress in the kidney manifested by elevated expression of renal Prdx2 [111]. Renal disease patients show significantly reduced plasma HDL-C, APOA-I, serum PON1 protein concentration, PON1 arylesterase/ paraoxonase activity, and LCAT activity [131], indicating the impaired interactions of PON1 with APOAI and LCAT in dysfunctional HDL in these patients.

Mice deficient in the scavenger receptor class B type I (*SR-BI*^−/−^) fed with a normal diet showed significantly reduced serum PON1 activity and renal PON1 expression, which was accompanied by renal pathology involving oxidative stress, inflammation, and fibrosis [116]. Western blot analysis and qRT-PCR showed that the expression of p47phox protein, a key regulator of NADPH oxidase, was significantly higher in the kidneys of *SR-BI*^−/−^ mice compared with wild type *SR-BI*^+/+^ mice. mRNA levels of NADPH oxidase genes *Nox 1* and *Nox4* were also significantly increased. Levels of inflammation-related Il1b and Il6 mRNAs were significantly increased, while anti-inflammatory cytokine Il10 mRNA tended to decrease. These findings show that the renal oxidative stress and inflammation were significantly upregulated in *SR-BI*^−/−^ mice compared to wild-type *SR-BI*^+/+^ animals.

Overexpression of PON1 (mRNA, 2.03-fold; protein, 3.36-fold) using a lentivirus vector significantly attenuated the pathologic changes in the kidneys of *SR-BI*^−/−^ mice fed with a high-fat diet. Specifically, PON1-overexpressing (Pon1^+^) *SR-BI*^−/−^ mice showed a significant decrease in the fluorescence intensity of dihydroethidium bromide staining, a significant decrease in the immunohistochemical renal staining for lipid peroxidation indicator 4-hydroxynonenal, a significant decrease in the oxidative stress-related indicators such as renal p47phox protein and Nox1, Nox2, Nox4 mRNAs, and a significant increase in the activity of the anti-oxidant enzyme SOD in the kidney. In addition, PON1-overexpressing mice showed reduced levels of *Tnf-α* and *Il6* mRNAs and elevated levels of anti-inflammatory cytokine Il10. These findings show that PON1 overexpression has beneficial effects on the kidney function in *SR-BI*^−/−^ mice by reducing renal ROS production, improving anti-oxidant status, and ameliorating renal inflammation. PON1 overexpression also attenuated renal lipid accumulation by upregulating cholesterol ester hydrolysis-related genes (*Nceh1*, *Lipa*) and cholesterol efflux-related receptors (Abca1, Abcag1). Western blot and qRT-PCR analyses showed that fibrosis-related proteins (fibronectin, collagen, type I, a1, and actin a2 in smooth muscle and aorta) were significantly downregulated in PON1-overexpressing mice compared to the control lentivirus-GFP-injected mice; mRNA levels for fibronectin, *Col1a1*, *Ccn2*, and *Lcn2*, a marker of kidney injury, were also significantly reduced, indicating that PON1 overexpression ameliorated fibrosis. Moreover, Pon1 overexpression inhibited mTOR signaling and restored autophagy flux in the mouse kidney [116]. That Pon1 can regulate mTOR signaling and autophagy was also reported in another study that found upregulated mTOR signaling and downregulated autophagy in *Pon1*^−/−^ mice brains and in *Pon1*-silenced mouse neural cells [15].

### 5.9. PON1-Q192R Polymorphism Influences Oxidative Stress and Inflammation Proteins in Human Plasma

**Figure 6:**
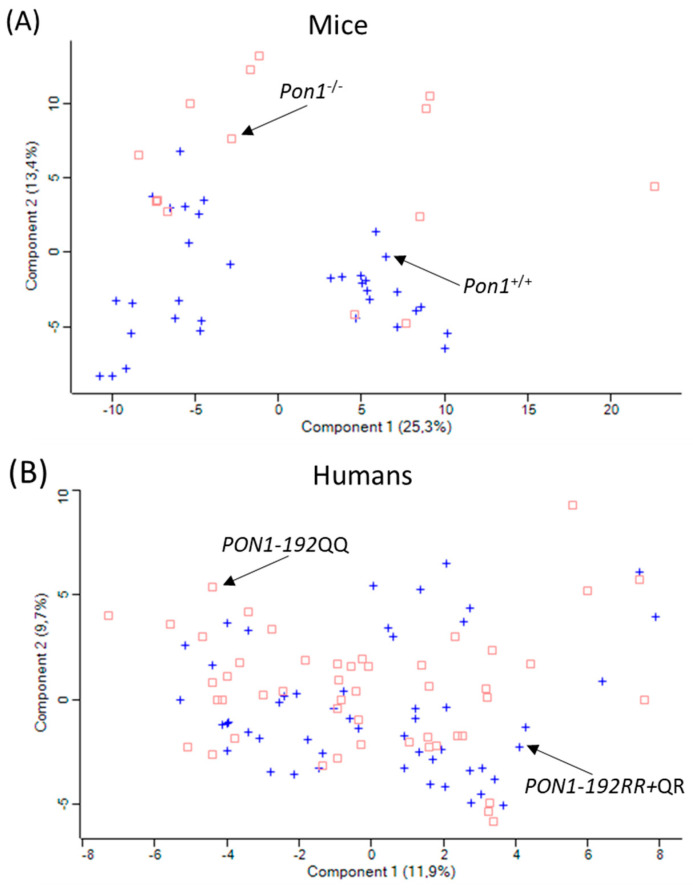
Principal component analysis of the LFQ intensities for plasma proteins. (**A**) *Pon1*^−/−^ mice (*n* = 17; blue cross) and *Pon1*^+/+^ siblings (*n* = 8; red square). (**B**). *PON1^−/−^-192QQ* (*n* = 50; blue cross) and *PON1^−/−^-192QQ* healthy humans (*n* = 50; red square). Calculations were performed with Perseus. Adapted from ref. [23].

To ascertain how changes in PON1 expression/activity affect the expression of other proteins, plasma proteomes were analyzed in healthy participants recruited from the population of Poznań, Poland [23]. *PON1-Q192R* polymorphism affected serum paraoxonase activity (7-fold reduction in *PON1-192QQ* vs. *PON1-192RR*) and protein (40% reduction *PON1-192QQ* vs. *PON1-192RR*) (Table 4). Label-free nanoLC-MS/MS mass spectrometry analyses showed that there was an overlap in the principal component analysis (PCA) profiles between low-activity *PON1-192QQ*, intermediate-activity *PON1-192QR,* and high-activity *PON1-192RR* genotypes (Figure 6B).

The *PON1-Q192R* polymorphism affected the expression of 21 plasma proteins, including six oxidative stress-related proteins (APOA1↓, PON1↓, APOD↑, APOM↑, haptoglobin (HP)↓, and glutathione peroxidase 3 (GPX3)↑), four immune response proteins: CFP↑, IGHG3↑, ↑PGLYRP2, and ↑V2-6 (IGL), four lipoprotein metabolism proteins (APOA1↓, APOB↑, APOC1↓, and PON1↓), two acute phase response protein (TTR ↑ and AMBP↑), and seven blood coagulation protein (F13B↓, PLG↓, SERPINA10↓, VTN↓, ↑C9, ↑V2-17 (IGL), and FETUB↑). Six of those proteins (APOA1, APOB, APOC1, APOD, APOM, and PON1), representing 29% of total number of proteins affected by the *PON1-Q192R* polymorphism, are components of HDL [10,132]. These findings suggest that PON1 regulates the expression of other components of HDL as well as HDL proteins involved in oxidative stress, inflammation, and complement/coagulation in humans. The dysregulation of these processes may account for the pro-oxidant and pro-atherogenic phenotypes associated with attenuated PON1 levels in humans [28] and mice [55].

### 5.10. Pon1^−/−^ Genotype Influences Oxidative Stress and Inflammation Proteins in Mouse Plasma

Pon1 activity and protein are absent in *Pon1*^−/−^ mice [55] (Table 5). To determine how Pon1 depletion affects the expression of other proteins, plasma proteomes were analyzed in *Pon1*^−/−^ mice (*n* = 17) and their *Pon1*^+/+^ littermates (*n* = 8) using label-free nanoLC-MS/MS mass spectrometry [23]. PCA profiles differed between *Pon1*^−/−^ vs. *Pon1*^+/+^ siblings (Figure 6A). Pon1 depletion in mice affected the expression of 50 plasma proteins, including seven redox proteins (↓Alb, ↓Blvrb, ↑ Ambp, ↑Hp, ↑Hpx, ↑ApoD, and ↑ApoM), seven lipoprotein metabolism proteins (↓ApoA1, ↑ApoB, ↓ApoC1, ↑ApoD, ↑ApoM, ↓Pon1, and ↑Lcat), four acute phase response proteins (↑Ambp, ↑Hp, ↑Hpx, and ↑Ttr), and 11 complement/coagulation proteins (↑Al182371, ↑Cfh, ↑Clu, ↑F2 (prothrombin), ↓Klkb1, ↓Mbl1, ↓Serpinc1, ↑Fetub, ↓F13B, ↑Hrg, and ↓Itih1) (arrows indicate direction of change). Nine of those proteins (↓Alb, ↓ApoA1, ↓ApoC1, ↑ApoD, ↑ApoM, ↑Clu, ↑Saa1, ↑Saa2, and ↑Lcat), representing 18% of total number of proteins affected by the *Pon1*^−/−^ genotype, are components of HDL [10,16]. Clu (ApoJ) is also involved in amyloid beta (Aβ) transport from plasma to the brain [76].

Nine proteins that were affected by Pon1 genotype in mice (↓ApoA1↓, ↑ApoD↑, ↑ApoM↑, ↓Pon1↓, ↑haptoglobin (Hp)↓, ↓Ighg3↑, ↑Ttr↓, ↓F13B↓, and ↑Fetub↓), were also affected by the *PON1-Q192R* polymorphism in humans (representing 22% and 43% of the total number of differentiating proteins in mice and humans, respectively) (right and left arrows refer to the change in humans and mice, respectively). These findings show that changes in the mouse plasma proteome associated with the *Pon1*^−/−^ genotype were like those in the human plasma proteome associated with the *PON1-Q192R* polymorphism. These findings also suggest that Pon1 regulates the expression of other protein components of HDL in addition to proteins involved in oxidative stress, inflammation, and complement/coagulation in mice.

**Figure 7:**
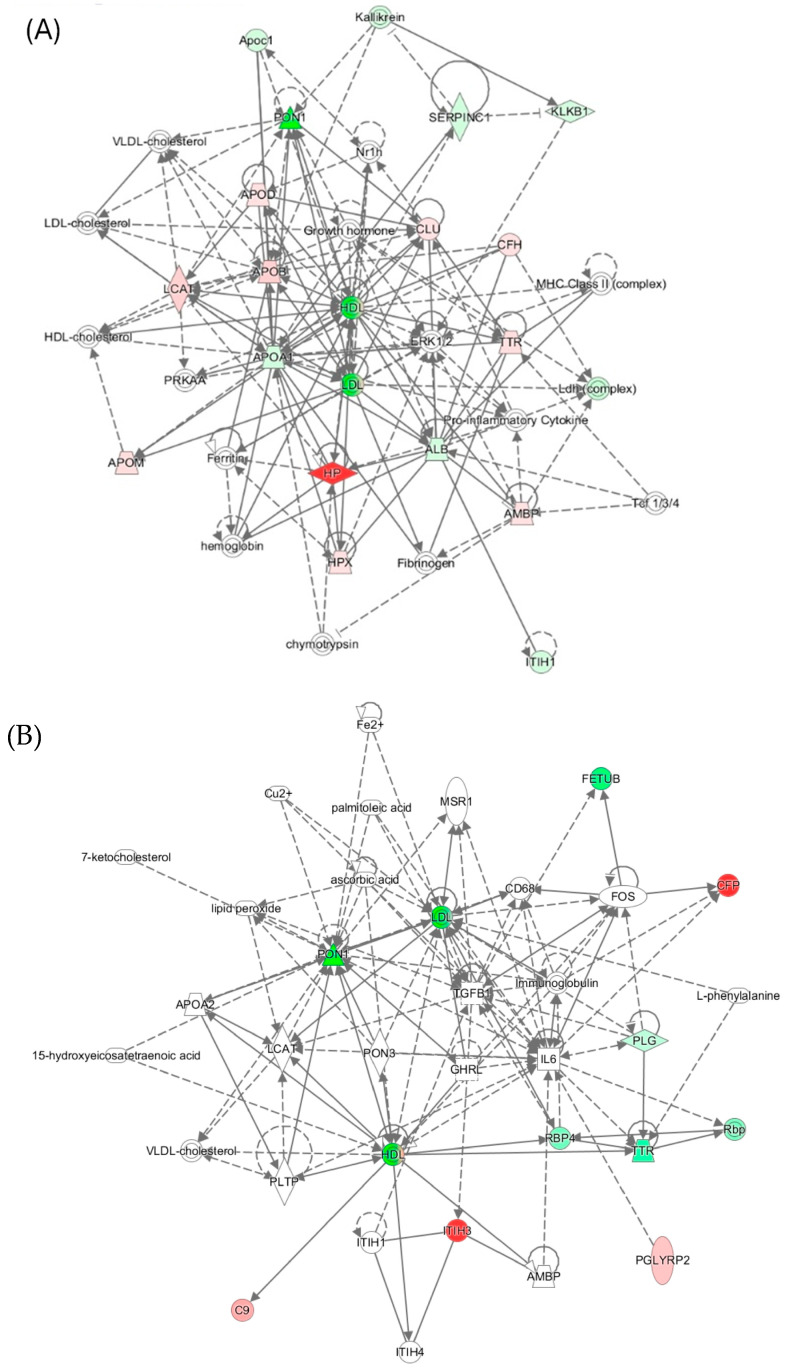
Top molecular network ‘Lipid Metabolism, Molecular Transport, Small Molecule Biochemistry’ associated with (**A**) *Pon1*^−/−^ genotype in mice and (**B**) *PON1-Q192R* polymorphism in humans. The mouse network (**A**) includes redox-related proteins (Alb, Ambp, Hp, Hpx, ApoD, and ApoM) and inflammation-related proteins (Ambp and Ttr). The human network (**B**) contains inflammation-related AMBP and TTR proteins. Reprinted with permission from ref. [23]. Copyright 2020 by the authors.

Ingenuity pathway analysis identified the “Lipid Metabolism, Molecular Transport, Small Molecule Biochemistry” network, affected by PON1 activity in mice (Figure 7A) and humans (Figure 7B). Proteins in the human network are involved in acute phase/immune response and lipid metabolism and exhibit strong interactions focusing on LDL and HDL, the cytokine IL6, and the transforming growth factor beta 1 (TGFB1). In mice, this network contains oxidative stress proteins such as Alb, Hpx, Hp, Blvrd, Ambp, ApoD, and ApoM.

These findings suggest that PON1 interacts with molecular pathways involved in oxidative stress, inflammatory response, complement/blood coagulation, and lipoprotein metabolism, processes that are essential for blood homeostasis. The dysregulation of these processes by attenuated PON1 protein/activity levels can account for PON1’s association with cardiovascular and neurological diseases and cancer.

### 5.11. Metabolic Stress Amplifies Pro-Inflammatory, Pro-Oxidative, and Pro-Atherogenic Changes in Mouse Plasma Proteome Induced by Pon1 Depletion

**Figure 8:**
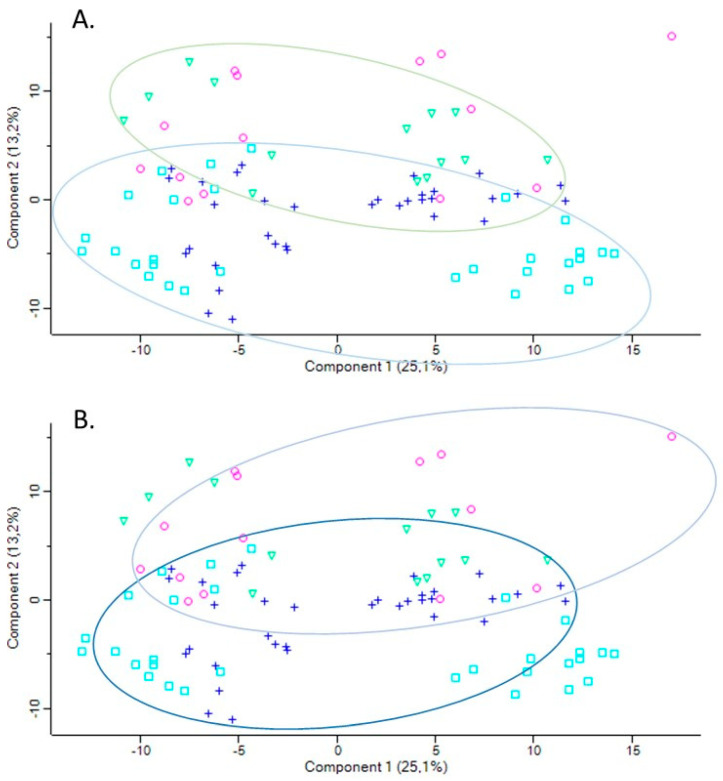
Principal component analysis (PCA) of label-free quantification (LFQ) intensities of mouse plasma proteins: effects of *Pon1* genotype and diet. *Pon1*^−/−^ mice, control diet (*n* = 17, blue cross); *Pon1*^−/−^ mice, HHcy diet (*n* = 15, blue square); *Pon1*^+/+^ siblings, control diet (*n* = 8, purple circle); *Pon1*^+/+^ siblings, HHcy diet (*n* = 7, green triangle). Calculations were carried out using Perseus. Ovals illustrate a smaller overlap between data points for *Pon1*^−/−^ and *Pon1*^+/+^ mice fed with high-met (**A**) than for mice fed with control diet (**B**). Reprinted with permission from ref. [114]. Copyright 2021 by Elsevier, Inc.

To determine the effects of Pon1 depletion under the condition of metabolic stress on gene expression, plasma proteomes were examined in *Pon1*^−/−^ mice and their wild-type *Pon1*^+/+^ littermates fed with a high-methionine HHcy diet [114]. There was a clear difference in the PCA profiles of LFQ intensities for *Pon1*^−/−^ mice compared to wild-type *Pon1*^+/+^ animals, with a partial overlap between them (seven out of 44, 16%; *Pon1*^−/−^, blue squares □ vs. *Pon1*^+/+^, green triangles ∆) (Figure 8). In mice fed with a standard chow diet, the overlap was greater (28 out of 48, 58%; *Pon1*^−/−^, blue crosses + vs. *Pon1*^+/+^, purple circles ○), indicating that the *Pon1*^−/−^ genotype exerts a stronger influence under the conditions of the metabolic stress of HHcy (Figure 8).

Pon1 depletion in HHcy mice changed the expression of 89 proteins, 1.8-fold more than in chow diet mice, including 18 redox proteins, 24 immune response proteins, six acute phase proteins, 15 complement/blood coagulation proteins, nine lipoprotein/lipid metabolism proteins, protein turnover proteins, and 10 other proteins (Table 6). Eight of those proteins (↓Alb, ↓ApoA1, ↓ApoA2, ↑ApoB, ↓ApoC1, ↓ApoC2, ↑Clu, and ↓Pon1), representing 8% of total number of proteins affected by the *Pon1*^−/−^ genotype, are components of HDL [10,132].

The largest changes in the number of *Pon1*^−/−^ genotype-dependent proteins in HHcy vs. chow diet mice were observed for oxidative stress-related proteins (18 proteins in HHcy diet vs. four proteins in chow diet mice), acute phase proteins (seven vs. two), and protein turnover proteins (six vs. two) (Table 6). Smaller changes between the diets were observed in the number of proteins involved in immune response (24 vs. 19), complement/coagulation (eight vs. seven), blood coagulation (six vs. three), lipoprotein/lipid metabolism (nine vs. eight), and other proteins (10 vs. five) (Table 6). These findings clearly show that the metabolic stress of HHcy greatly amplifies the effects of the *Pon1^−/−^* genotype on oxidative stress and inflammation.

Eleven of the proteins (12%) affected by the *Pon1*^−/−^ genotype in HHcy mice (ApoA1, ApoA2, ApoC1, ApoC2, Pltp, Saa1, Saa2, Alb, A2m, Pros1, and Pon1) are the components of HDL [10,130], some of which were found to be enriched in the PON1-containg HDL subfraction (Alb, Clu, A2m, and Pros1) [16]. Phospholipid transfer protein (Pltp), found to be upregulated in *Pon1*^−/−^ HHcy mice (Table 6), regulates the size/composition of HDL in the circulation and controls plasma HDL levels [133]. These findings show that Pon1 affects the expression of plasma proteins involved in oxidative stress, inflammation, and other processes linked to CVD. The dysregulation of these processes may account for the pro-oxidant and pro-atherogenic phenotypes associated with attenuated PON1 levels in humans [28] and mice [55].

Nineteen oxidative stress-related proteins such as Parkinson disease protein 7 (Park7, DJ-1), peroxiredoxin-2 (Prdx2), peroxiredoxin-6 (Prdx6), and thioredoxin (Txn) were significantly downregulated, while seven inflammatory response proteins were upregulated in HHcy Pon1-depleted mice (Table 6). The impairment of anti-oxidant and anti-inflammatory defenses caused by changes in the expression of oxidative stress- and inflammation-related proteins can account for the increased oxidative stress and inflammation observed in *Pon1*^−/−^ mice [55] and in humans with low activity of PON1 [28].

## 6. Conclusions

Transcriptomic and proteomic analyses provided new insights regarding PON1 function by identifying the proteins and molecular pathways influenced by PON1 depletion or PON1 overexpression. Accumulating evidence shows that changes in PON1 expression/activity influence both extracellular and cellular proteostasis by impairing epigenetic regulation, upregulating mTOR signaling, and inhibiting autophagy. Pon1 depletion induces oxidative stress and inflammation by influencing the expression of genes involved in these processes. The changes in gene expression caused by low PON1 expression/activity levels are exacerbated by the metabolic stress of hyperlipidemia or hyperhomocysteinemia. Although these changes are linked to CVD, Alzheimer’s disease, and cancer, the molecular mechanisms by which PON1 affects gene expression remain to be elucidated in future studies.

## Figures and Tables

**Table 1:** Association of PON1 with oxidative stress, inflammation, and human disease *.

Disease	PON1 Activity	*PON1* Genotype	Oxidative Stress	Inflammation	References
Cardiovascular disease	↓	*PON1-192QQ*	↑	↑	[28,30,57,58]
Kidney disease	↓	ND	ND	↑	[31,32]
Fatty liver disease	↓	ND	↑	↑	[59]
Alzheimer’s disease	↓	*PON1-107TT*	↑	ND	[33,60,61,63]
Hepatocellular carcinoma	↓	ND	↑	↑	[34,62]

* ND, not determined. Up and down arrows indicate the direction of change.

**Table 2:** Brain proteins affected by *Pon1* depletion and/or HHcy in mice are also affected in AD and other neuropathies.

Protein Name	Change in *Pon1^−/−^* vs. *Pon1^+/+^* Brain *		Change in Human AD Brain (Another Neuropathy or Animal Model) **
	Std. Diet	1%-Met Diet	
*Brain-specific*			
Ncald	–	↑	↓, (↓ *Gls*^−/−^ mouse)
Nrgn	↓	↑	↓
Stmn1	–	↑	↓, (↑ MS, TLE, SMA, schizophrenia), (↑ HD4 mouse model)
*Anti-oxidant defense*			
Sod1	↓	–	(↑ ALS)
Prdx2	–	↑	↑
DJ-1 (Park7)	↓	↑	↑
*Energy metabolism*			
Ak1	–	↑	↑
*Cell cycle*			
GDI1	–	↑	(↑ rat ischemic brain)
Ran	–	↑	↑
*Cytoskeleton assembly*			
Tbcb	↓	↑	(↑ GAN)
CapZa2	↑	–	↑ CapZb2 ^#^
*Other proteins*			
Hdhd2	–	↑	

* The up ‘**↑**’ and down ‘**↓**’ arrows indicate up-regulated and down-regulated proteins, respectively. No significant change is indicated by dash ‘–‘. ** AD, Alzheimer’s disease; MS, multiple sclerosis; HD, Huntington disease; GAN, giant axon neuropathy; TLE, temporal lobe epilepsy; SMA, spinal muscular atrophy. ^#^ The b2 subunit of the CapZ heterodimer. Adapted from ref. [60].

**Table 3:** PON1 influences oxidative stress- and inflammation-related gene expression.

Model	Treatment	Gene Expression Assessment		Outcome		References
		Methods	Tissue	In Vivo	In Vitro	
*Pon1*^−/−^ vs. *Pon1*^+/+^mice, high fat diet	Pon1 deletion in WT mice	Enzymatic activity assay, Northern blot, RT-qPCR, Western blot	Liver, plasma	Pon1 protein absent in *Pon1*^−/−^ mice, ↑lipid peroxides in HDL, no lipid peroxides in LDL, ↑atherosclerosis; no atherosclerosis in *Pon1*^−/−^ mice on a chow diet	↑Lipid peroxides in hLDL, ↑MCP1, ↑monocyte migration ameliorated by *Pon1*^+/+^ HDL in a cell co-culture model of the arterial wall; Pon1^−/−^ HDL does not prevent hLDL oxidation	Shih D.M. et al. [55]
*Pon1*^−/−^*ApoE*^−/−^ vs. *Pon1*^+/+^*ApoE*^−/−^ mice, std chow diet	Pon1 deletion in *ApoE*^−/−^ mice	Enzymatic activity assay, Northern blot, RT-qPCR	Liver, plasma	↑Lipid peroxides in LDL, ↑oxidized phospholipid epitopes in plasma; ↑HO1, PPARγ, ↑oxLDL receptors (SRA, CD36, macrosialin), but not HDL receptor SR-BI, in the liver; no change in anti-oxLDL and anti-MDA-LDL autoantibody levels; ↑atherosclerosis	LDL from *Pon1*^−/−^ mice elevated lipid hydroperoxide and monocyte transmigration	Shih D.M. et al. [56]
Human *PON* cluster transgenic mice	*PON1*, *PON2*, *PON3* overexpression	Enzymatic activity assay, RT-qPCR, Western blot, ELISA		↑PON1 expression, ↓atherosclerosis ↓Icam-1, ↓Mcp-1, ↓TNF-α, ↓IL-6, ↓Mmp-9	PON Tg HDL ameliorated Cu-induced LDL oxidation	She Z.G. et al. [66]
*Pon1*^−/−^ vs. *Pon1*^+/+^mice, std chow diet	Pon1 deletion	2D-SDS-PAGE gel electrophoresis, MALDI-TOF mass spectrometry	Brain	↓Sod1, ↓DJ-1		[111] Brain
			Liver	↑Prdx2, ↑Ftl, ↑ApoE		[112] Liver
			Kidney	↑Prdx2, ↓ApoA1		[113] Kidney
		Enzymatic activity assay, label-free nanoLC-MS/MS mass spectrometry	Plasma	↓Alb, ↓Blvrb, ↑Ambp, ↑(Hpx, ↑ApoD, ↑ApoM, ↑Hp), ↑Ttr		Sikora M. et al. [23]
*Pon1*^−/−^ vs. *Pon1*^+/+^mice, high methionine diet	Pon1 deletion	Enzymatic activity assay, label-free nanoLC-MS/MS mass spectrometry	Plasma	↓**Alb**, ↑Hp, ↑Hpx, ↓Alad, ↑Cp, ↓Gclm, ↓Cat, ↑Ctsb, ↓Gsn, ↑Grn, ↓Prdx2 ↓Prdx6, ↓Txn, ↓Igfbp3, ↓Park7, ↓Pebp1, ↓Ppia, ↓Serpina3k		Sikora M. et al. [114]
*PON1-192QQ* vs. *PON1-192RR+QR* humans	none	Enzymatic activity assay, label-free nanoLC-MS/MS mass spectrometry	Plasma	APOA1↓, PON1↓, APOD↑, APOM↑, HP↓, GPX3↑		Sikora M. et al. [23]
*SR-BI*^−/−^ vs *SR-BI*^−/−^ mice	*SR-BI* deletion, probucol treatment	Enzymatic activity assay, 2D-SDS-PAGE gel electrophoresis, LC-MS/MS mass spectrometry, Western blot, RT-qPCR	Plasma, HDL	↓HDL protein, ↓ApoA1, ↓Pon1 protein and activity, ↑Saa, ↑ApoA4, ↑A1AT, ↑Mpo Probucol upregulated Pon1, downregulated Saa, ApoA4, A1AT, and Mpo thereby improving HDL function	↑Mcp1, Tnf-α in oxLDL treated macrophages; *SR-BI*^+/+^ HDL reduced Mcp1, Tnf-α while *SR-BI*^−/−^ HDL had no effect	Cao J. et al. [115]
*Pon1^+^SR-BI*^−/−^ mice	Pon1 overexpression *SR-BI*^−/−^ mice using lentivirus vector	Enzymatic activity assay, RT-qPCR, Western blot	Kidney	*Pon1^+^SR-BI*^−/−^ mice: ↓renal Pon1 expression and plasma activity, ↑ expression of redox (p47phox, Nox1, Nox4) and inflammation related (Il1b, Il6) genes, ↓anti-inflammatory cytokine Il10. *Pon1^+^SR-BI*^−/−^ mice fed with a high-fat diet: ↑plasma and renal Pon1 expression and activity, ↓renal redox (p47phox, Nox1, Nox4, Sod) and inflammation related (Tnfα, Il6) genes, ↑anti-inflammatory cytokine Il10		Liu Q. et al. [116]
			Liver	*Pon1^+^SR-BI*^−/−^ mice: ↑hepatic/plasma Pon1, ↑ApoE, ↑Lcat, ↓plasma ALT activity, ↓ROS levels and MPO activity, ↓acute-phase and pro-inflammatory plasma proteins (ApoA4, A1AT, Saa), ↑hepatic ApoA1, Ldlr, Lxrα, Abca, Abcg5, Abcg8, ↓Tnf-α, ↓Il6, ↓ atherosclerosis	Macrophages treated with *Pon1^+^SR-BI*^−/−^ HDL: ↓mRNA for inflammatory cytokines IL-6, TNFα, NOX1, ↑mRNA for anti-inflammatory cytokines IL-4, IL-10; mRNA for cholesterol transport ↓Scarb1, ↑Abca1	Zhao X.J. et al. [117]

HO1, heme oxidase 1; PPARγ, peroxisome proliferator-activated receptor gamma; MCP1, monocyte chemotactic protein; MPO, myeloperoxidase; *Pon1^+^SR-BI*^−/−^ mice, Pon1-overexpressing *SR-BI*^−/−^ mice; Up and down arrows indicate the direction of change in gene expression.

**Table 4:** *PON1* genotype, activity, and protein levels in humans.

Human PON1		
Genotype (n)	Activity ^a^	Protein ^b^
*PON1-192RR* (19)	100	100
*PON1-192QR* (30)	20.6	63.0
*PON1-192QQ* (51)	14.3	60.0

^a^ Relative mean values determined in serum using paraoxon as a substrate. ^b^ PON1 protein quantified by label-free mass spectrometry. Adapted with permission from ref. [23]. Copyright 2020 by the authors.

**Table 5:** *Pon1* genotype, activity, and protein levels in mice.

Mouse Pon1		
Genotype (n)	Activity ^a^	Protein ^b^
*Pon1*^+/+^ (17)	100	100
*Pon1*^−/−^ (8)	0.0	2.0

^a^ Relative mean values, determined in serum using paraoxon as a substrate. ^b^ PON1 protein quantified by label-free mass spectrometry. Adapted from ref. [23].

**Table 6:** Plasma proteins affected by *Pon1^−/−^* genotype in mice fed with HHcy or control diet *.

Unique to HHcy-Diet Mice (*n* = 66)	Unique to Control-Diet Mice (*n* = 27)	Proteins Affected Both in HHcy and Control-Diet Mice (*n* = 23)
Oxidative stress (*n* = 15): ↓Alad, ↑Cp, ↓Gclm, ↓Cat, ↑Ctsb, ↓Gsn, ↑Grn, ↓Prdx2 ^#^, ↓Prdx6, ↓Txn, ↓Igfbp3, ↓Park7 ^#^, ↓Pebp1 ^#^, ↓Ppia, ↓Serpina3k	Oxidative stress (*n* = 1): ↓Blvrb	Oxidative stress (*n* = 3): ↓**Alb** ^$^, ↑Hp, ↑Hpx
Immune response (*n* = 15): ↓Il1rap, Igh (*n* = 10↑, 1↓), ↑Igk (*n* = 3),	Immune response (*n* = 10): ↑**Clu** ^$^, ↑Igh (*n* = 3↑, 1↓), ↑Igk (*n* = 3), ↑Igl, ↑Igm	Immune response (*n* = 9): ↑Igh (*n* = 4), ↑Igj, ↑Igk (*n* = 3), ↑Igl
Acute phase response (*n* = 6): ↑**A2m** ^$^, ↑Ahsg, ↑Orm1, ↑Orm2, ↑**Saa1**, ↑**Saa2**	Acute phase response (*n* = 1): ↑Ttr	Acute phase response (*n* = 1): ↑Ambp
Complement/coagulation (*n* = 5): ↑Apcs, ↓F13a1, ↑C3, ↑Cfb, ↑Cfhr1	Complement/coagulation (*n* = 4): ↑AI182371, ↑F2, ↓F13b, ↓Mbl1	Complement/coagulation (*n* = 3): ↑Cfh, ↓Klkb1, ↓Serpinc1
Blood coagulation (*n* = 6): ↑Serpina10, ↓Gp1ba, ↑Gp5, ↑Itih3, ↑**Pros1** ^$^, ↓Proz	Blood coagulation (*n* = 3): ↓Hgfac, ↑**Hrg** ^‡^, ↓Itih1	
Lipoprotein/lipid metabolism (*n* = 5): ↓**ApoA2**, ↓**ApoC2**, ↓Azgp1, ↓Pgp, ↑**Pltp**	Lipoprotein metabolism (*n* = 4): ↓Afm, ↑**ApoD**, ↑**ApoM**, ↑**Lcat**	Lipoprotein metabolism (*n* = 4): ↓**ApoA1**, *↑***ApoB**, ↓**ApoC1**, ↓**Pon1**
Protein turnover (*n* = 5): ↓Apeh, ↓Mug2, ↓Serpina3m, ↓Uba1, ↓Uba52	Protein turnover (*n* = 1): ↑Fetub	Protein turnover (*n* = 1): ↓Mug1
Other proteins (*n* = 8): ↓Atic, ↓Nme1, ↓Pnp (purine metabolism), ↓Tpi, ↓Tkt (glucose metabolism), ↓Ran ^#^ (nucleoplasmic transport), ↓Rbp4 (retinol transport), ↓Spp2 (bone remodeling)	Other proteins (*n* = 3): ↓Aldoa, ↓Ldha (glucose metabolism), ↓Lifr (tissue regeneration)	Other proteins (*n* = 2): ↓Bpgm (glucose metabolism), ↓Ica (carbonic anhydrase inhibitor)

* Up and down arrows indicate the direction of change in protein levels. ^#^ Proteins affected also in mouse brain [58]. ^$^ Enriched in, or ^‡^ largely excluded from, PON1-containg HDL subfraction of normal human HDL [16]. Bold font indicates proteins identified as components of HDL. Adapted with permission from ref. [114]. Copyright 2021 by Elsevier, Inc.
